# Divergent extremes but convergent recovery of bacterial and archaeal soil communities to an ongoing subterranean coal mine fire

**DOI:** 10.1038/ismej.2017.1

**Published:** 2017-03-10

**Authors:** Sang-Hoon Lee, Jackson W Sorensen, Keara L Grady, Tammy C Tobin, Ashley Shade

**Affiliations:** 1 Department of Microbiology and Molecular Genetics, Michigan State University, East Lansing, MI, USA; 2 School of Civil, Environmental, and Architectual Engineering, Korea University, Seoul, South Korea; 3 Department of Biology, Susquehanna University, Selinsgrove PA, USA; 4 Program in Ecology, Evolutionary Biology, and Behavior, Michigan State University, East Lansing, MI, USA

## Abstract

Press disturbances are stressors that are extended or ongoing relative to the generation times of community members, and, due to their longevity, have the potential to alter communities beyond the possibility of recovery. They also provide key opportunities to investigate ecological resilience and to probe biological limits in the face of prolonged stressors. The underground coal mine fire in Centralia, Pennsylvania has been burning since 1962 and severely alters the overlying surface soils by elevating temperatures and depositing coal combustion pollutants. As the fire burns along the coal seams to disturb new soils, previously disturbed soils return to ambient temperatures, resulting in a chronosequence of fire impact. We used 16S *rRNA* gene sequencing to examine bacterial and archaeal soil community responses along two active fire fronts in Centralia, and investigated the influences of assembly processes (selection, dispersal and drift) on community outcomes. The hottest soils harbored the most variable and divergent communities, despite their reduced diversity. Recovered soils converged toward similar community structures, demonstrating resilience within 10–20 years and exhibiting near-complete return to reference communities. Measured soil properties (selection), local dispersal, and neutral community assembly models could not explain the divergences of communities observed at temperature extremes, yet beta-null modeling suggested that communities at temperature extremes follow niche-based processes rather than null. We hypothesize that priority effects from responsive seed bank transitions may be key in explaining the multiple equilibria observed among communities at extreme temperatures. These results suggest that soils generally have an intrinsic capacity for robustness to varied disturbances, even to press disturbances considered to be ‘extreme', compounded, or incongruent with natural conditions.

## Introduction

Human interactions with and alterations of environmental systems are important components of global change (Allen *et al.*, 2014). Anthropogenic disturbances are outcomes of human activity, and include land use and land cover changes, pollution, dispersal of invasive species, and over-harvesting of native animal or plant populations (Vitousek *et al.*, 2008). Anthropogenic disturbances are typically classified as press disturbances, as they often impact multiple generations of organisms within their ecosystems (Bender *et al.*, 1984). Because of their longevity, press disturbances have the capacity to alter ecosystems beyond the possibility of recovery (for example, Thrush *et al.*, 2009).

Within every ecosystem, microbial communities underpin biogeochemical processes, sustain the bases of food webs, and recycle carbon and nutrients. In some situations of anthropogenic disturbance, such as pollution, native microbial communities also can provide bioremediative functions to support ecosystem recovery (Ruberto *et al.*, 2009; Desai *et al.*, 2010; Fuentes *et al.*, 2015; Ma *et al.*, 2016). Because of their foundational roles in driving important ecosystem processes, understanding how microbial communities respond to press disturbance can provide insights into the potential for ecosystems to recover. It may also help to uncover mechanisms by which environmental microbial communities may be managed to improve ecosystem outcomes. A better understanding of microbial responses to press disturbances, including examples of communities that have recovered or shifted to an alternative stable state, is necessary to move toward the goal of microbial community management (Shade and Peter *et al.*, 2012).

Recent work has highlighted the importance of understanding the relative contributions of community assembly processes to community changes (for example, Vellend, 2010; Ferrenberg *et al.*, 2013; Nemergut *et al.*, 2013; Vellend *et al.*, 2014; Dini-Andreote *et al.*, 2015; Evans *et al.*, 2016; Tucker *et al.*, 2016), and these processes can also be informative for understanding community changes after a disturbance (for example, secondary succession; Dini-Andreote *et al.*, 2015). According to Vellend, 2010, community assembly can be summarized by four major processes: dispersal, diversification, drift, and selection. *Dispersal* is the movement of individuals between localities, *diversification* is the generation of new genetic variation (which can lead to speciation), *drift* encompasses the stochastic processes resulting in fluctuations in member abundances (for example births and deaths), and *selection* refers to deterministic fitness differences among taxa driven by abiotic conditions or biotic interactions (as summarized by Nemergut *et al.*, 2013). Together, these processes complement and interact to drive community patterns, and together provide a foundation on which to build a predictive theoretical framework for microbial community ecology.

Because diversification processes are relatively more important at evolutionary scales, Vellend *et al.*, 2014 focused on the remaining processes of ecological selection, drift, and dispersal. They asserted that selection processes are deterministic, that drift processes are stochastic, and that dispersal processes can be either or both, depending on the situation (Vellend *et al.*, 2014). Tucker *et al.* (2016) provided clarity to the distinction between deterministic/stochastic and niche/neutral processes, which are often used interchangeably. Niche/neutral refers to the ecological differentiation and equivalence of species, while deterministic/stochastic refers to non-probabilistic or probabilistic outcomes (Tucker *et al.*, 2016). Thus, neutrality concerns ecological equivalence of species, while stochasticity concerns demographic variability in birth, death, and dispersal.

We aimed to understand the responses of soil microbial communities to an anthropogenic press disturbance, and to apply the Vellend, 2010, Nemergut *et al.*, 2013, and Tucker *et al.*, 2016 conceptual frameworks of community assembly for interpretation of patterns. The town of Centralia, Pennsylvania is the site of an underground coal mine fire that has been burning since 1962. It is one of thousands of coal mine fires burning in the world today (Melody and Johnston, 2015), which are inconspicuously common anthropogenic disturbances. However, the Centralia fire is especially long-lived, and, after efforts to extinguish it failed, it was left to burn until it self-extinguished (Nolter and Vice, 2004). The fire is expected to burn slowly until the coal reserves have been consumed. The fire currently underlies more than 150 acres and continues to spread slowly (3–7 m per year, Elick, 2011) through underground coal seams. Depending on the depth of the coal bed, it burns at an estimated 46-69 m below the surface (Nolter and Vice, 2004; Elick, 2011). Heat, steam and combustion products vent upward from the fire through the overlying soils. The surface soil temperatures can exceed 80 °C, scarring the landscape with dead vegetation that reveals the fire's subsurface trajectory. As steam and gasses pass through the overlying rock and soil, soil temperatures increase while soil chemical composition is altered by both spontaneous and microbial-mediated chemical reactions (Janzen and Tobin-Janzen, 2008). As the fire expands into new areas, it also retreats from some affected sites, which then recover to ambient temperatures (Nolter and Vice, 2004; Elick, 2011). Thus, the ‘end' of the disturbance can be delineated by temperature recovery. In this way, a chronosequence of fire-affected Centralia soils provides a space-for-time proxy of disturbance response and recovery.

Our research objectives were to understand the diversity and spatio-temporal dynamics of the surface soil bacterial and archaeal communities that have been impacted historically or are currently influenced by the ongoing subterranean coal mine fire in Centralia. We used a definition of disturbance response to include changes in member relative abundances as well as in composition. Previous work using terminal restriction fragment length polymorphism analysis showed that microbial diversity decreased at hotter sites, and that compositional changes were correlated with soil ammonium and nitrate concentrations (Tobin-Janzen *et al.*, 2005). We move forward from this work to use high throughput sequencing of soil community 16S rRNA genes to quantify the community dynamics along a chronosequence of fire response and recovery. We specifically investigated the community assembly processes of selection, dispersal, and drift.

## Materials and methods

### Study site, soil sampling, soil biogeochemistry and microbial community DNA extraction

We undertook fieldwork in Centralia (GPS: 46°46”24'N, 122°50”36 W) on 5–6 October 2014. We collected surface soils to capture the expected maximum changes along a chronosequence of fire recovery (Supplementary Figure 1). We sampled two fire fronts along gradients of historical fire activity. Fronts are trajectories of fire spread from the 1962 ignition site outward along near-surface coal seams (Elick, 2011). These fronts include surface soils that were previously hot and have cooled, as well as soils that are currently warmed by the ongoing fire. We collected soil from two unaffected, proximate sites as references, seven recovered sites along the gradient, and nine fire-affected sites (18 total soils), and these collections were distributed across both fire fronts. Soil samples were collected from the top 20 cm of surface soil (core diameter 5.1 cm), and were sieved through 4 mm stainless steel mesh. We collected cores only at bare surface soil locations (no vegetation) to minimize the influence of local vegetation and to maximize comparability between soils, as the thermal surface soils generally lacked vegetation. Collected soils were stored on ice up to 72 h during transport to the laboratory, then stored at −80 °C pending further processing. The physico-chemical characteristics of each soil sample (percent moisture, organic matter (500 °C), NO_3_^-^, NH_4_^+^, pH, SO_4_, K, Ca, Mg, P, As, and Fe) were assayed by the Michigan State Soil and Plant Nutrient Laboratory according to their standard protocols (East Lansing, MI, USA, http://www.spnl.msu.edu/). Gravimetric soil moisture was measured after drying the soil at 80 °C for 2 days. Fire history was estimated as years since the surface soil was first hot from the fire, at each sampling location. Fire history observations were measured using either winter snow cover, aerial vegetation photography, or thermal infrared imagery, as collated and reported by Elick, 2011 (Figure 3 therein). Soil community DNA was extracted from 0.25 g of soil in three technical replicates using the MoBio Power Soil DNA Isolation Kit according to the manufacturer's protocol (MoBio, Solana Beach, CA, USA). The concentration of the extracted DNA was measured using the Qubit dsDNA BR Assay Kit (Life Technologies, NY, USA), and DNA amount was standardized for sequencing to 1,000 ng/sample.

### Soil cell counts

Direct bacterial and archaeal cell counts were conducted on frozen soil samples based on a protocol to separate cells from soil reported in (Portillo *et al.*, 2013). To dissociate the microbial cells from soil particles, 10 g of soil was mixed with 100 ml of phosphate buffered saline containing 0.5% Tween-20 (PBST). Soil samples were homogenized in a Waring blender three times for 1 min each, followed by a 5 min incubation on ice. Slurries were centrifuged at 1000 × *g* for 15 min to concentrate soil particulates. Supernatants were set aside and stored at 4 °C, and the remaining soil pellets were re-suspended in 100 ml of fresh PBST and blended for an additional 1 min. The soil slurry was then transferred to sterile 250 ml centrifuge bottles and the blender was washed with an additional 25 ml of sterile PBST and added to the slurry before centrifugation at 1000 × *g* for 15 min. All resulting supernatants for each site were combined, then centrifuged at 10 000 × *g* for 30 min to pellet cells. Supernatants were discarded, and cell pellets were re-suspended in 10 ml of sterile Milli-q water and 400 ml of 37% formaldehyde to fix cells. 1 ml of cell suspension was then carefully layered over 500 μl of sterile Nycodenz solution (0.8 g ml^−1^ in 0.85% NaCl), then centrifuged at 10 000 × *g* for 40 min. The upper layer was then collected and cells were pelleted by centrifugation at 20 000 × *g* for 15 min, then re-suspended in 1 ml of sterile 0.85% NaCl. To dissociate remaining soil clumps, cell suspensions were sonicated for 10 s in a sonicating water bath.

Cell suspensions were stained with DTAF ((5-(4,6-Dichlorotriazinyl) Aminofluorescein)) according to (Robertson *et al.*, 1999). DTAF-stained smears were visualized on a Nikon Eclipse e800 microscope (Tokyo, Japan) equipped with a Photometrics Coolsnap Myo camera (Tuscon, AZ, USA), and images were collected using Micro-Manager software (Edelstein *et al.*, 2014). Fiji image analysis software was used to adjust background, thresholding, and to conduct particle counts from images (Schindelin *et al.*, 2012). Briefly, background correction was completed using an automated rolling ball subtraction with a 35-pixel radius, followed by automatic local thresholding using the Bernsen method with a 12-pixel radius to convert greyscale images to binary. Watershed segmentation was conducted to separate touching nuclei, then particles were counted using the ImageJ ‘Analyze Particles' function, excluding anything smaller than 0.1 micron (Schneider *et al.*, 2012).

### Quantitative PCR

We performed quantitative PCR (qPCR) using bacterial and archaeal 16S rRNA gene universal primer sets (Supplementary Table 1; Caporaso *et al.*, 2012). The reaction mixtures consisted of 10 μl SYBR qPCR Master mix (Quanta Bioscience, Gaithersburg, MD, USA), 0.4 μl each of the forward and the reverse primers (0.4 pm), 2 μl of template DNA, and sterilized deionized water to adjust the final volume of 20 μl. The thermal profile was as follows: initial denaturation at 95 °C for 10 s, followed by 40 cycles of denaturation at 95 °C for 10 s, annealing at 50 °C for 15 s, and extension at 72 °C for 40 s. A final dissociation protocol (58–94.5 °C, increment 0.5 °C for 10 s) was performed to ensure the absence of nonspecific amplicons. The reactions were conducted using the Bio-Rad iQ5 real time detection system (Bio-Rad, Hercules, CA, USA). Please see the Supplementary materials for more details as to the qPCR methods.

### 16S rRNA amplicon sequencing

For each of the 54 DNA samples (18 soils, each with three replicate DNA extractions) and mock community DNA, paired-end sequencing (150 base pair) was performed on the bacterial and archaeal 16S *rRNA* gene V4 hypervariable region using the Illumina MiSeq platform (Illumina, CA, USA; Supplementary Table 1; Caporaso *et al.*, 2012). All of the sequencing procedures, including the construction of Illumina sequencing library using the Illumina TruSeq Nano DNA Library Preparation Kit, emulsion PCR, and MiSeq sequencing were performed by the Michigan State University Genomics Core sequencing facility (East Lansing, MI, USA) following their standard protocols. The Genomics Core provided standard Illumina quality control, including base calling by Illumina Real Time Analysis v1.18.61, demultiplexing, adaptor and barcode removal, and RTA conversion to FastQ format by Illumina Bcl2Fastq v1.8.4. Raw sequences were submitted to the GenBank SRA Accession SRP082686.

To estimate sequencing error, mock community DNA was prepared from six different type strains (*D. radiodurans* ATCC13939, *B. thailandensis* E264, *B. cereus* UW85, *P. syringae* DC3000, *F. johnsoniae* UW101, *E. coli* MG1655). The genomic DNA from these type strains were extracted separately using the EZNA Bacterial DNA Kit (Omega Bio-tek, GA, USA) according to the manufacturer's protocol, and then quantified using the Qubit dsDNA BR Assay Kit (Life Technologies, NY, USA). Each isolates' 16S rRNA sequence was amplified using universal 27F and 1492R primers. Amplification was performed with the GoTaq Green Master Mix (Promega) with the following reaction conditions: 0.4μm each primer, 20–200 ng template, 12.5 μl 2 × GoTaq Green Mastermix and nuclease free water to 25 μl final volume. The products were visualized on 1% agarose gels before being cleaned using the Promega Wizard SV Gel and PCR Cleanup System per manufacturer's instructions. Cleaned amplification products were sequenced using the 27F and 1492R primers using the ABI Prism BigDye Terminator Version 3.1 Cycle kit at Michigan State's Genomics Research Technology Support Facility (https://rtsf.natsci.msu.edu/genomics/). Forward and reverse reads were merged using the merger tool in the EMBOSS (V. 6.5.7) package (Rice *et al.*, 2000). Based on the DNA concentration, size of genomic DNA, and 16S rRNA gene copy number, the final mixture contained 100 000 copies of 16S rRNA gene from each strain. The mock community was sequenced alongside the 54 soils' metagenomic DNA. All sequences are available in NCBI's Short Read Archive (https://www.ncbi.nlm.nih.gov/sra/SRP082686).

### Sequence processing

Paired-end sequence merging, quality filtering, denoising, singleton-sequence removal, chimera checking, and open-reference Operational Taxonomic Unit (OTU) picking were conducted using a UPARSE workflow v8.1 (Edgar, 2013; Edgar and Flyvbjerg, 2014). Open-reference OTU picking was modified for compatibility with the UPARSE pipeline but proceeded as described for open-reference workflows (Rideout *et al.*, 2014). We selected open-reference OTU picking because it allowed us to retain all high-quality sequences, even if they did not match to the reference database. In addition, we expected novel diversity in Centralia, and it was likely that many Centralia sequences would not hit to reference databases. Furthermore, we wanted to create consistent OTU definitions that could be tractable across this study and future work. In the open-reference OTU picking workflow, reference-based OTU clustering first was conducted using the usearch_global command to cluster sequences with 97% identity to the greengenes database (v 13.8, http://greengenes.secondgenome.com/downloads). Second, de novo OTU picking was performed for any sequences that did not hit the greengenes reference; the usearch command cluster_otus was used to cluster sequences at 97% identity (this step includes chimera checking). The reference-based and de novo OTUs were combined together to create the final data set. Finally, to reduce the potential effects of candidate contaminant sequences, any sequences in the final data set that matched 100% to a database of extraneous sequences (found in the mock community) were removed.

Additional analyses were performed with QIIME v.1.9.1 (Caporaso *et al.*, 2010b), including alignment with PyNAST (Caporaso *et al.*, 2010a), taxonomic assignment with the RDP Classifier (Wang *et al.*, 2007), tree building with FastTree (Price *et al.*, 2009), subsampling/rarefaction to an equal sequencing depth, and within and comparative diversity calculations (for example, UniFrac, Lozupone and Knight, 2005). Sequences identified as Chlorophyta, Streptophyta (that is, Chloroplasts) and Mitochondria were removed before subsampling to an even sequencing depth. Our sequence analysis workflow and computing notes are available on GitHub (https://github.com/ShadeLab/PAPER_LeeSorensen_ISMEJ_2017/tree/master/Sequence_analysis). We used the UPARSE workflow (with the recommended 10% divergence filter) for error rate calculation using the mock community (http://drive5.com/usearch/manual/upp_tut_misop_qual.html).

### Ecological statistics

We first assessed the reproducibility of evenly-sequenced technical replicates (DNA extraction and sequencing replicates), and found that replicates were similar to one another in measures of within-sample (alpha) and comparative diversity (beta diversity). The average and standard deviation of weighted non-normalized UniFrac distances between replicates was 0.319±0.126 with a range from 0.105 to 1.29 (maximum distance between different samples was 4.49; Supplementary Figure 2; and alpha diversity among technical replicates provided in Supplementary Table 2). Given the low technical variability, unrarefied technical replicates were collapsed into one combined set of sequences for each soil core to provide more exhaustive sequencing of each soil; these collapsed samples were subsampled to an even sequencing depth (321,000 sequences per soil), and singleton OTUs (observed only once in the data set) were removed before proceeding with analysis. Within-sample diversity of species richness, Faith's phylogenetic diversity (whole tree method), and comparative diversity of weighted and unweighted UniFrac distance (non-normalized and normalized, (Lozupone *et al.*, 2007, 2011) were calculated within QIIME. Non-normalized UniFrac distances can fall outside of 0 and 1, while normalized UniFrac distances are bound to 0 to 1; Lozupone *et al.*, 2007 reported no differences in overarching patterns in beta diversity between the non-normalized and normalized UniFrac (Lozupone *et al.*, 2007), and we have found that this holds for our data set (Supplementary Table 3). The data were then moved into the R environment for statistical analyses. Briefly, we used vegan functions for multivariate hypothesis testing, fitting environmental vectors to ordinations (envfit), constrained ordination (capscale), and Mantel tests (mantel) and to calculate Pielou's evenness (Oksanen *et al.*, 2011); the cmdscale function (stats) for principal coordinates analysis; custom code of neutral models of community assembly (Sloan *et al.*, 2007) as written and implemented by Burns *et al.*, 2015 (‘sncm.fit_function.R'); custom R scripts for beta-null model fitting written by Tucker *et al.*, 2016, Appendix 2 therein) modified by our group to include weighted UniFrac beta-null modeling; and ggplot and ggplots2 for plotting (Wickham, 2009). Our R script is available on GitHub (‘R_analysis' repository in https://github.com/ShadeLab/PAPER_LeeSorensen_ISMEJ_2017/tree/master/R_analysis).

## Results and discussion

### Soil physical-chemical characteristics and microbial population size

We measured a suite of contextual data for each sampling site, and asked whether any of those data were correlated with surface soil temperature (Supplementary Figure 3). Centralia soils generally represented a wide range of soil chemistry. We did not find strong correlations between measured contextual data and temperature, with the exception of correlations with ammonium and nitrate (Pearson's R=0.50 and 0.54, respectively; *P*<0.05). This finding supports previous work in Centralia showing that ammonium and nitrate were elevated at active vents (Tobin-Janzen *et al.*, 2005). In addition, the pH of recovered sites was consistently lower than reference sites (mean pH=4.4 and 5.9, respectively), and the hottest soils were more likely to have extreme or disparate values. In two previous reports, soil ammonium, nitrate, and sulfur concentrations were not necessarily correlated with absolute soil temperature values at Centralia, nor to proximity to an active vent; though extreme or disparate chemistry values were sometimes observed at hot sites, values comparable to unaffected sites were also routinely observed (Tobin-Janzen *et al.*, 2005; Janzen and Tobin-Janzen, 2008). The authors suggested that duration of fire impact, whether the fire was advancing or receding from the site, and other complex environmental factors were likely contributing.

All soils were within one order of magnitude of 16S rRNA copies per dry mass of soil with fire-affected soils having the highest copy numbers and recovered soils having the lowest, but there were no statistical differences among groups (Supplementary Figure 4A, Student's t-test all pairwise p⩾0.09). Total number of cells per dry mass of all soil ranged from 10^5^ to 10^7^ cells per gram of dry soil, but cell counts across fire classifications also were not statistically distinct (Supplementary Figure 4B, Student's *t*-test all pairwise p≥0.09). Together, these data indicate overall community size is relatively stable across the fire gradient and that any changes in community structure along the fire gradient are due to changes in member abundances rather than to differences in the total number of individuals (community size) among soils.

Sequencing efforts were near-exhaustive for these soils, as assessed by a clear asymptote achieved with rarefaction (Supplementary Figure 5). A summary of sequencing efforts, as well as a discussion of reference-based and *de novo* OTU taxonomic assignments for fire-affected and recovered soils, are provided in Supplementary materials.

### Selection

**Figure 1:**
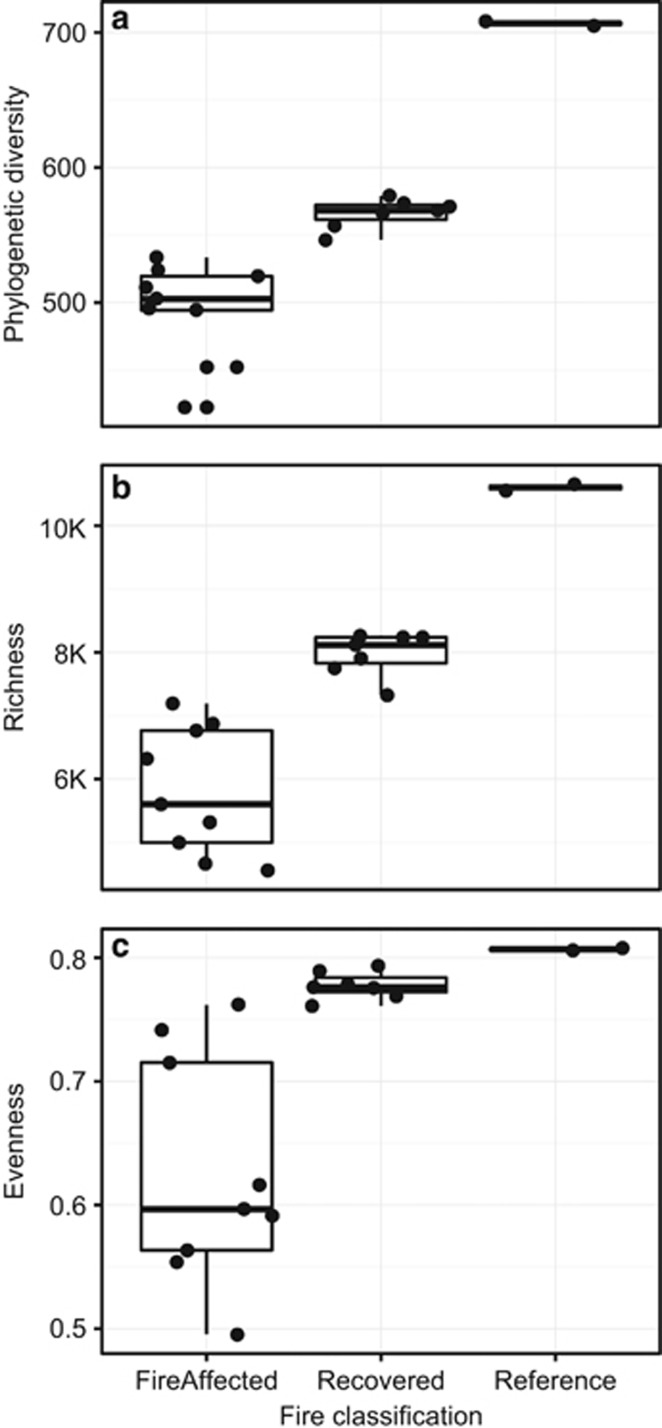
Within-sample (alpha) diversity of fire-affected, recovered, and reference soils in Centralia for bacterial and archaeal community (**a**) Faith's phylogenetic diversity (all *P*<0.001); (**b**) richness (total no. observed OTUs clustered at 97% sequence identity, all *P*<0.001); and (**c**) Pielou's evenness (all *P* not significant).

To understand the influence of selection (deterministic) processes on community responses, we used surface soil temperatures measured in 2014 to designate categorical groups of communities according to their fire classification. Soils classified as reference and recovered had temperatures between 12 °C and 15 °C (ambient air temperature was 13.3 °C at the time of soil collection), while soils classified as fire-affected had temperatures ranging from 21 °C to 58 °C. We hypothesized that within-sample diversity would be lower in fire-affected soils because of the extreme environmental filter of high temperatures, which we expected to result in lower richness and less phylogenetic breadth. Faith's phylogenetic diversity and OTU richness both were lowest and most variable for fire-affected soils, and highest for reference sites (Figure 1; Student's *t*-test all pairwise *P*<0.001). Pielou's evenness had a similar trend, with fire-affected soils having lower evenness than other soils, suggesting that there are a small number of highly dominant OTUs in the fire-affected soils (all pairwise *P*>0.05, not significant). These results generally agree with studies investigating soil microbial diversity after coal mine reclamation in China and Brazil, respectively, where the most recovered/reconstructed soils (20 years post-mining in Li *et al.*, 2014) and 19 years of reconstruction in de Quadros *et al.*, 2016) had highest within-sample diversity and were most comparable to reference sites. Centralia soils are expected to share similar contamination from coal extraction with these mine reclamation soils, but also are distinct because of their thermal conditions and ongoing surface contamination by coal combustion products, such as inorganic gases containing arsenic, selenium, ammonium, sulfur, and hydrogen sulfide, and organic toxins like polycyclic aromatic hydrocarbons (Janzen and Tobin-Janzen, 2008). Elements within inorganic gases mineralize and deposit around active vents (Janzen and Tobin-Janzen, 2008). Some coal combustion products, like volatile sulfur and nitrogen compounds, may enrich for microorganisms capable of using them, while other combustion products, like organic toxins, may decrease microbial community size or diversity (Janzen and Tobin-Janzen, 2008).

**Figure 2:**
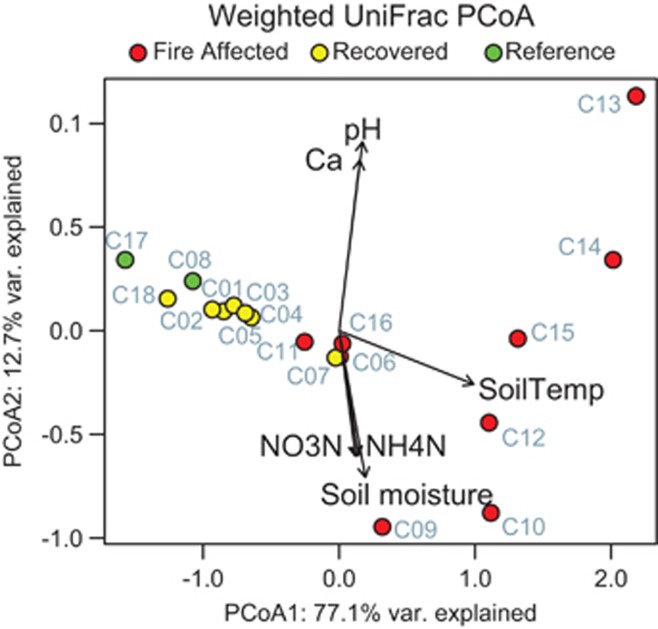
Principal coordinate analysis (PCoA) based on weighted UniFrac distances of phylogenetic bacterial and archaeal community structure. Colors show the fire classification of the soil as fire-affected (red), recovered (yellow), or reference (green). The strength of statistically significant (*P*<0.10) explanatory variables are shown with solid arrows.

We used weighted UniFrac distance to assess comparative community diversity across the fire categories. Weighted UniFrac distance was chosen after considering multiple taxonomic and phylogenetic, and weighted and unweighted metrics. All resemblances revealed the same overarching patterns (all pairwise Mantel and PROTEST *P*<0.001, Supplementary Table 3), demonstrating that these patterns were very robust. However, weighted UniFrac distance provided the highest explanatory value (Supplementary Table 3), suggesting that changes in both phylogenetic breadth and the relative abundances of taxa are important for interpreting community responses. As compared to recovered and reference sites, fire-affected soils were distinct (PERMANOVA pseudo F=16.10, *R*^2^=0.50 and *P*=0.001 on 1000 permutations) and more variable in their community structure (difference in median dispersions=0.53, *P*=0.008; Figure 2). Differences in surface soil temperature had most explanatory value on Axis 1 (77.1% variance explained by Axis 1, temperature Axis 1 correlation=0.97, *P*=0.001, Supplementary Table 4), with nitrate and iron contributing; calcium and pH (and, to a lesser extent, soil moisture) explained variation on Axis 2 (12.7% variance explained by Axis 2, Supplementary Table 4). Notably, soil fire history (estimated years since the local soil surface was first measured hot as reported by Elick, 2011) was not correlated to community dynamics (Supplementary Table 4).

Fire-affected soils were more variable in their community structure across soils, especially in soils at the most extreme temperatures observed (sites C13, C10 which were >50 °C at the time of sampling and were at the opposite ends of PCoA2). In contrast, recovered soils were less variable, even though they spanned decades of difference in their years of peak fire activity (the earliest impacted soils that we sampled were last recorded to be hot in 1980; Elick, 2011). Also, recovered soils were very similar in community structure to reference soils. These patterns show that Centralia soils achieve divergent community structures over the transition from ambient to extreme conditions, but then generally converge towards a consistent community structure after the fire subsides. These results also show resilience of soil communities impacted by an extreme press disturbance, with recovery occurring within 10–20 years after the stressor subsided.

We observed a temperature ‘threshold' effect among fire-affected soils, and soils with temperatures between 21 °C and 24.5 °C (sites C06, C11 and C16) separated cleanly from soils with temperatures greater than 30 °C (Figure 2). To better understand the divergence in community structure among fire-affected soils, we performed a PCoA with these communities (Supplementary Figure 6A, Supplementary Table 5), and also a constrained analysis to ask what variability remained after removing the influence of temperature (Supplementary Figure 6B, Supplementary Table 6). Even after removing the influence of temperature, three discrete subsets of fire-affected communities separated from each other along both axes, with C13 remaining as an outlying point. C13 had very different calcium and pH than the other soils, and both of these factors had high value in discriminating C13 from the other fire-affected soils (*P*=0.092 and 0.014, respectively). There were no other measured abiotic factors that explained the divergence among the fire-affected soils. In addition, the constrained axes had high explanatory value (Supplementary Figure 6B, combined axes 1 and 2=90.0% var. explained), suggesting that, given the measured conditions, there are additional processes beyond abiotic selection that explain the differences in these subsets.

**Figure 3:**
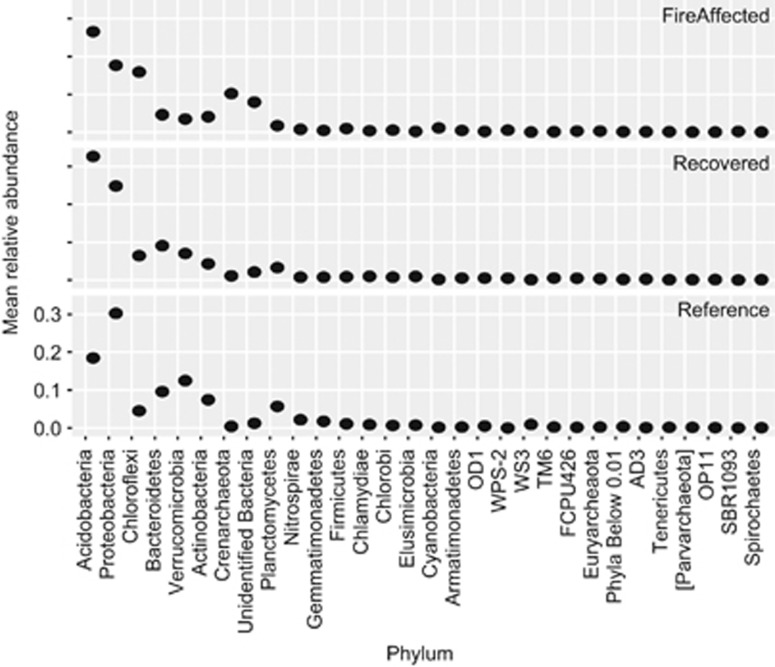
Phylum-level responses to the Centralia coal mine fire. Mean relative abundance of phyla summarized within soil fire classifications (fire-affected, recovered, and reference). Unidentified Bacteria are a combination of OTUs unable to be assigned taxonomy at the phylum level, and are not a monophyletic group. ‘Phyla Below 0.01' are all OTUs assigned to phyla that collectively comprise less than 0.01 relative abundance and also are not a monophyletic group.

We observed broad phylum-level changes in response to the fire (Figure 3, Supplementary Table 8). Not all OTUs affiliated with particular phyla had identical responses; however, our analysis of phylum-level responses points to some general trends. In particular, fire-affected soils were enriched for members of Chloroflexi, Crenarcheaota and many lineages of unidentified Bacteria. As compared to the fire-affected soils, recovered soils also were enriched for Parvarchaeota, Bacteroidetes, Elusimicrobia, Gemmatimonadetes, Planctomycetes, Spirochaetes, TM6, and Verrucomicrobia suggesting that members affiliated with this these phyla are able to persist after the fire subsides. Acidobacteria also had an increase in recovered soils (but less significant, *P*=0.10), presumably because of the decrease in soil pH observed post-fire (Supplementary Figure 3, pH panel: row 1, column 3). Reference soils had higher representation of Proteobacteria and Verrucomicrobia, which suggests that members of these phyla may be sensitive to the fire.

### Dispersal and drift

To investigate the relative importance of local dispersal, we assessed the value of spatial distance for explaining differences in community structure. If local dispersal were important, we would expect that soils in close proximity would have more similar community structures than soils that are distant from one another. We found no relationship in the measured spatial distances between soil collection sites and their corresponding differences in community structure for all sites (Mantel *P*=0.66 on 999 permutations), nor for recovered sites only (after removing the fire-affected sites from analysis; Mantel *P*=0.135 on 999 permutations). The lack of evidence for spatial autocorrelation suggests that local dispersal is not a key factor shaping community structure in Centralia soils.

To explore the relative importance of drift in fire-affected and recovered soils, we used two complementary approaches. First, we fitted a neutral model of community assembly. The model predicts taxon frequencies as a function of their metacommunity log abundances, which is one method to consider the influence of drift with the influence of dispersal (calculated as an immigration term, *m*, to the model). The neutral model fit better to the recovered sites than to fire-affected sites (*R*^2^=0.53, 0.12, respectively; Supplementary Figure 7, Supplementary Table 7). Furthermore, we found a lower influence of dispersal (lower value of *m)* in the fire-affected sites (Supplementary Table 7). These differences in fit and generally minimal influence of dispersal suggest that neutral processes play a more minor role in the microbial community assembly of fire-affected sites than they do in the recovered sites.

**Figure 4:**
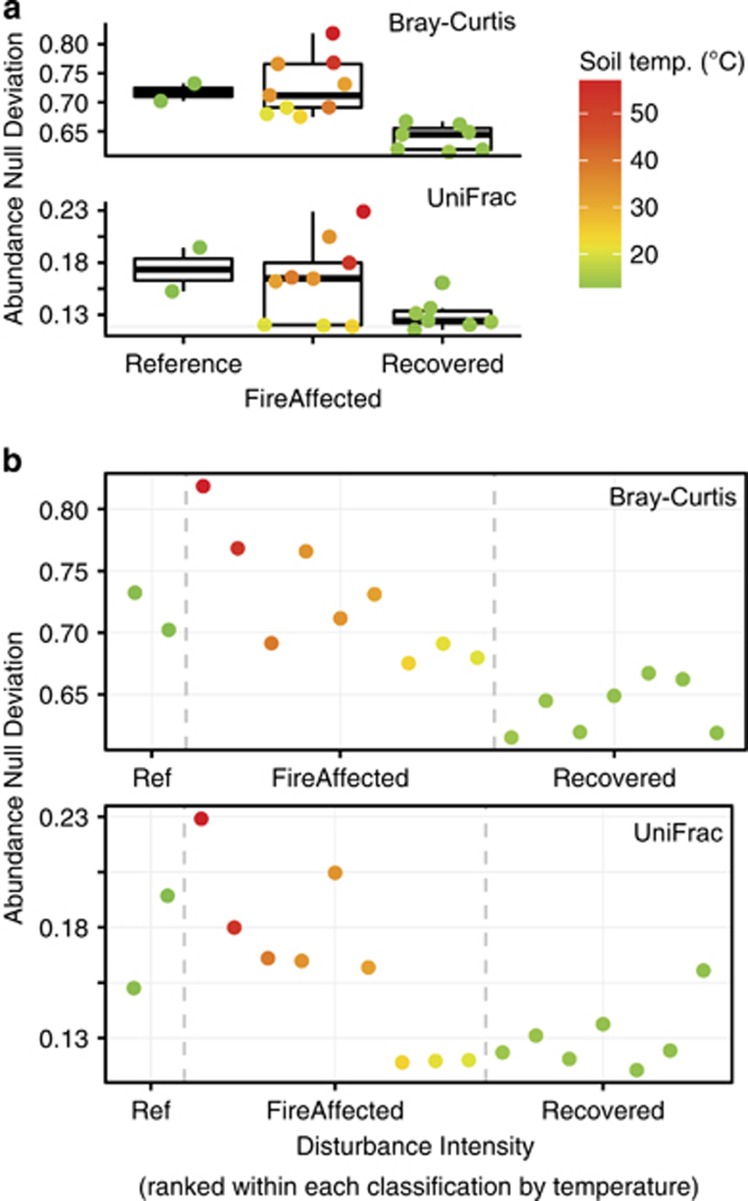
The relative changes in niche and neutral processes assessed using deviations from abundance-weighted beta-null models. Color gradient shows the soil temperature, as a proxy for disturbance intensity. (**a**) Abundance null deviations by fire classification. For both Bray-Curtis and weighted Unifrac resemblances, recovered and fire-affected communities had distinct null deviations (both *P*<0.05); (**b**) Trajectory of beta-null deviations ranked by disturbance intensity from reference to fire-affected to recovered soils. Weighted UniFrac and Bray-Curtis trajectories are correlated (*P*=0.71, *P*=0.001).

Next, we asked how observed differences in beta diversity deviate from null expectations. We used abundance-based beta-null approaches to distinguish niche and null processes according to Tucker *et al.*, 2016, and we extended their approach to also consider community differences in phylogenetic breadth by applying it to weighted UniFrac distances. In this comparative approach, deviations to and from a permuted null expectation (neutral) are used to interpret the relative influences of neutral and niche processes, respectively. All Centralia communities deviated from neutral, with reference and recovered soils falling closer to neutral expectations than fire-affected soils (Figure 4a). Fire-affected soils had statistically higher beta-null deviations than recovered soils (both *P*<0.05 for Bray-Curtis and weighted UniFrac). In the fire-affected soils, there was a consistent increase in niche processes with increasing soil temperature, and the hottest sites deviated furthest from the neutral expectation (Figure 4b). Accounting for phylogenetic breadth (using weighted UniFrac distance, Figure 4b suggested relatively less deviation from neutral than accounting for abundance alone (using Bray-Curtis dissimilarity, Figure 4b), but both resemblances had similar trends (Pearson's *R*=0.71, *P*=0.001) and produced identical statistical outcomes. These abundance null deviation results agree with the Sloan neutral model because they suggest that unmeasured niche processes structure soil communities at temperature extremes.

### Understanding community divergences at temperature extremes

To dig deeper into the differences in the three subsets of fire-affected soil (Supplementary Figure 6) that were not well explained by measured abiotic selection, local dispersal, or drift as assessed by the Sloan neutral model of community assembly and beta-null modeling, we asked if there were notable differences in their dominant memberships. Fire-affected soils generally had more variability and greater phylogenetic breadth in their dominant membership than recovered soils, and each fire-affected subset harbored an exclusive membership among their most prevalent taxa. We examined the top 10 prevalent taxa from each of the nine fire-affected soils. Collectively, there were 68 unique top 10 OTUs in fire-affected soils (out of a possible 90, if each of the nine fire-affected soil harbored mutually exclusive membership across their top 10). These prevalent fire-affected OTUs spanned fourteen phyla or Proteobacteria classes, included 30 *de novo* OTUs, and included seven taxa of unidentified Bacteria and two taxa of unidentified Proteobacteria. Acidobacteria OTUs were detected among the top 10 for all fire-affected soils, and eight of nine fire-affected soils included Chloroflexi among the top 10 OTUs. In comparison, recovered soils included ten phyla or Proteobacteria classes among their collective top 10, had no unidentified Bacteria or Proteobacteria, and included four *de novo* OTUs. Acidobacteria and Alphaproteobacteria OTUs were among the top 10 for all recovered soils, and six of the seven recovered soils also included Deltaproteobacteria. Together, these results show that fire-affected soils were more divergent and diverse in their prevalent membership than recovered soils.

**Figure 5:**
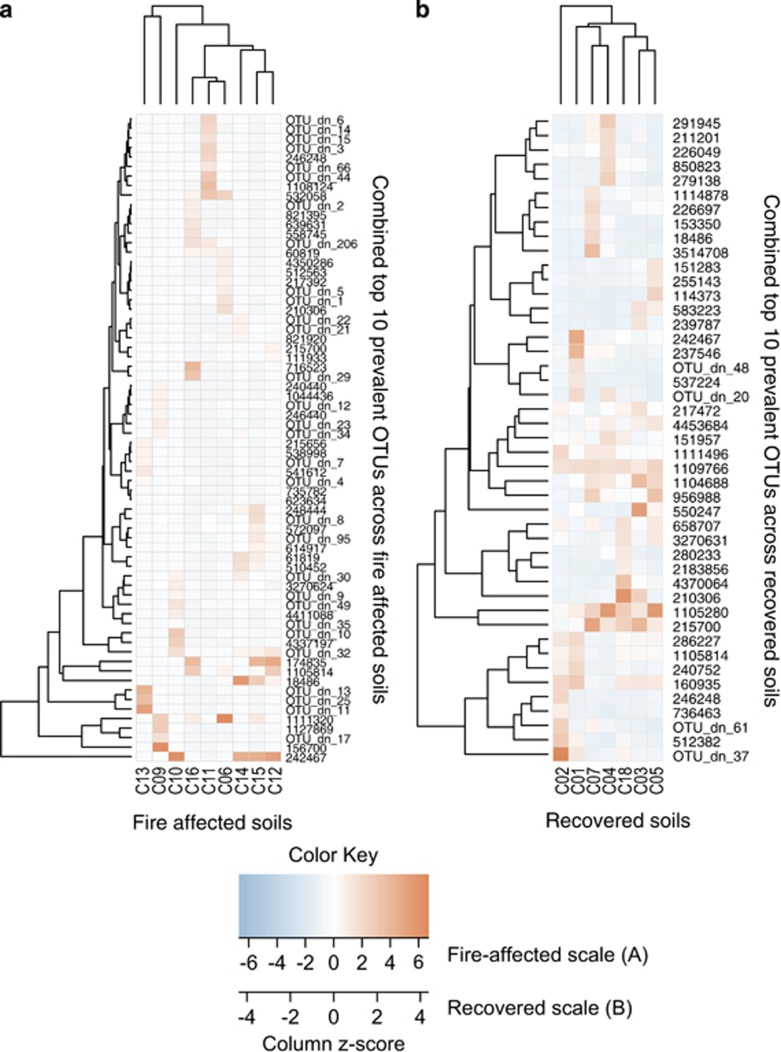
Relative abundances of the collection of the most prevalent combined ‘top 10' taxa (rows) observed in (**a**) fire-affected or (**b**) recovered soils (columns) in Centralia. Color gradients indicate taxa relative abundances, with warm colors indicating prevalent taxa and cool colors indicating rare taxa within that soil. Note differences in color scale gradient between **a** and **b**. Column labels are sample IDs, and OTU IDs are provided as row labels. OTU IDs that begin ‘OTU_dn' indicate that the taxon was clustered *de novo* in the open-reference OTU picking workflow; IDs that are numeric indicate that the taxon was assigned with high identity to a reference in the greengenes database. For reference-based OTUs, the numeric identifier corresponds to its representative sequence in the greengenes database. Top dendrograms cluster soils that have similar community structure, and side dendrograms cluster OTUs that have similar occurrence patterns.

An analysis of occurrence patterns of prevalent OTUs also showed greater divergence among fire-affected soils than recovered (Figure 5), and further supported the distinction among the subsets of fire-affected soils revealed by the constrained ordination (Supplementary Figure 6B). Fire-affected soils had more OTUs within their collective most prevalent taxa, and were more heterogeneous as shown by the wider range represented by the color scale and the more divergent sample and OTU clustering. In fact, taxa that were among the top 10 in one fire-affected soil were likely to be among the rare biosphere in another fire-affected soil, exhibiting stark contrast in their abundances within these soils. However, most of the top 10 prevalent OTUs were detected within every fire-affected soil (Table 1, Figure 5), suggesting that changes in taxa relative abundances, rather than turnover in membership, were driving these patterns.

This dominance analysis helps to explain the lower fit of the neutral model, and the relatively higher influence of niche processes with beta-null modeling, to fire-affected communities. Outliers to the neutral model that were below detection (taxa that were present in fewer sites than predicted given their relative abundance in the metacommunity) included these many lineages that were prevalent in few fire-affected soils. Taxa that fall below their neutral model prediction have been proposed to be ‘selected against' or particularly dispersal limited (Burns *et al.*, 2015). However, in the Centralia extreme environment, we suggest these are taxa that were most successful locally given the thermal disturbance.

### Community assembly processes given a press disturbance

Centralia soil communities were sensitive to the coal mine fire, and changed substantially from reference conditions. Selection processes, specifically abiotic soil conditions, offered high explanatory value for Centralia soil community dynamics. These communities first were constrained by environmental filters imposed by the press disturbance, such as thermal temperatures in fire-affected soils and low pH in recovered soils. The fire acts as a strong environmental filter, resulting in decreased diversity and a very different phylogenetic representation among the surviving lineages in fire-affected soils. These environmental filters, such as changes in pH, likely alter the functions of the community as well as its composition. However, even after removing the influence of temperature on fire-affected communities, the communities fell into three distinct subsets that could not be explained by the physico-chemical characteristics measured. Furthermore, neutral modeling, beta-null modeling and lack of spatial autocorrelation suggests that these particular assessments for drift and dispersal processes offer minimal explanation for fire-affected sites. Given the low explanatory value of unweighted resemblances in describing patterns of comparative diversity (Supplementary Table 3), and the observation that many of the prevalent taxa detected in some fire-affected soils were rare in other fire-affected soils (Figure 5a), we can also attribute these patterns to changes in the relative abundances of taxa within a locality, rather than to changes in taxa turnover (differing memberships). Thus, given that neither assessed selection, dispersal, nor drift processes, nor their combination can provide a complete explanation for the divergence of fire-affected communities, the questions remain: why are fire-affected soils so divergent from each other, and how do they eventually manage to recover to the same post-disturbance community structure?

One hypothesis is that the remaining variability in community structure of fire-affected sites may be attributed to priority effects initiated from different local transitions between the dormant seed bank and the active community. The proportion of dormant cells in soils is estimated to be as high as 80% (Lennon and Jones, 2011), and the importance of dormancy for microbial community assembly processes has been discussed at length (Nemergut *et al.*, 2013). Specific to the Centralia coal mine fire disturbance, thermophiles are prime examples of microbial seed bank members that often have been found in environments that are improbable to permit their growth (for example, McBee and McBee, 1956; Hubert *et al.*, 2009; Portillo *et al.*, 2012).

There are two aspects of seed banks that could help to explain Centralia community divergences at temperature extremes: membership and dynamics. If each soil harbored a different seed bank membership, different thermophilic taxa could become active and prevalent in each fire-affected soil, and would manifest as drift influences. This scenario is not well-supported by our data because we detect the dominant members of each fire-affected soil in the other fire-affected soils, albeit in lower abundances. Alternatively, awakenings from the microbial seed bank (Buerger *et al.*, 2012) could result in priority effects at temperature extremes, in which the first fit microorganisms to wake after the fire's local onset have important influence over the community's ultimate trajectory (for example, Fukami, 2015). In our chronosequence study, the outcome of priority effects would appear as divergent community structures at high temperatures that are explained by niche processes. In addition, unknown nuances in local abiotic conditions at fire onset could also set communities onto parallel trajectories and result in multiple equilibria during the press, which would also be explained by niche processes. Our data indirectly support either of these last two scenarios, as the three separate clusters of fire-affected communities suggest multiple equilibria (Supplementary Figure 6B). It could be that the most similar fire-affected communities began either from the same (or functionally equivalent) waking pioneer taxon, or from the same abiotic conditions (that are similar beyond reaching thermal temperatures), or from some combination of both, which initiated distinct trajectories towards each equilibrium.

Diversification is a fourth community assembly process discussed by Vellend, 2010 and Nemergut *et al.*, 2013. At ecological time scales, diversification was suggested by Vellend *et al.*, 2014 to have relatively lower influence than the other community assembly processes. We do not directly address diversification in this study, focusing instead on ecological processes. Aside from a consistent observation of Acidobacteria and Chloroflexi among the dominant taxa in fire-affected soils, there is no evidence that different but closely related lineages are most prevalent across all fire-affected soils, which may have hinted at distinct but parallel trajectories of diversification within a locality. However, we cannot reject the hypothesis that diversification processes also contribute to divergences in community structure at temperature extremes.

### Conceptual model

Extending the conceptual models of Ferrenberg *et al.*, 2013; Dini-Andreote *et al.*, 2015, we present a hypothesis of the assembly processes shaping communities before, during, and after an extreme press disturbance. Our model is based on our chronosequence trajectory for beta-null data presented in Figure 4b, and includes a phase encompassing the press disturbance, which extends beyond the representation of a pulse disturbance as a single time point as typical in previous conceptual models. Our model also incorporates a hypothesis of multiple transient equilibria within the press disturbance phase. We apply the advice of Tucker *et al.*, 2016 to not use the direction of the change from neutral (positive or negative) to infer specific ecological processes.

**Figure 6:**
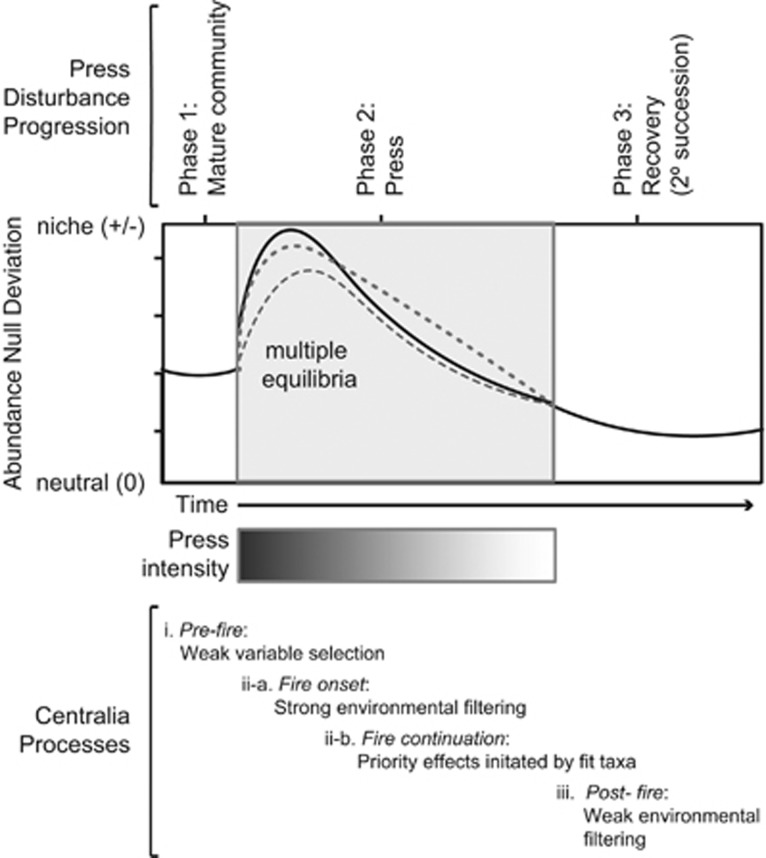
Hypothesized conceptual model of Centralia community assembly following press disturbance. Phase 1 represents the stable soil community pre-fire, and is characterized by weak variable selection from typical soil heterogeneity and high community diversity. Because the disturbance is a press, phase 2 occurs concurrent with the fire, when strong environmental filters (homogenizing selection) imposed by the extreme conditions drive a sharp increase in niche processes away from neutral conditions at the onset of the fire. Within phase 2, multiple equilibria result from priority effects of pioneer taxa that are fit to survive in the extreme press environment. Phase 3 is post-fire, characterized by relatively weak environmental filtering (for example, increased in soil acidity) that relaxes communities towards neutral. Complete neutrality was not observed in pre-fire or post-fire soils.

We hypothesize that weak variable selection drives stability in heterogeneous Centralia soil communities before the fire (reference sites in Figure 4; phase 1 in Figure 6). This is additionally supported by the literature demonstrating generally high heterogeneity and diversity in mature soil microbial communities (for example*,* O'Brien *et al.*, 2016). Next, strong environmental filtering from thermal temperatures (homogeneous selection, phase 2) decreases community diversity at the onset of the press disturbance. The lower diversity and prolonged disturbance conditions permit priority effects initiated by taxa fit in the thermal environment (for example, thermophiles waking from the seed bank), which set communities onto distinct deterministic trajectories with multiple equilibria during the fire (phase 2). Alternatively, the distinct trajectories and multiple equilibria could have been initiated by unmeasured nuances in abiotic conditions at thermal onset. Finally, weak environmental filtering from increased soil acidity relaxes communities back towards neutral in post-fire conditions (homogeneous selection, phase 3).

Regardless of the interim dynamics that resulted in community divergence to the stressor, Centralia soils eventually recovered to a community structure very similar to reference soils, and these community structures were explained by the ultimate post-fire soil environment. Our results show that Centralia soil communities, though sensitive to this extreme, complex, and arguably unnatural stressor, had near-complete return to pre-disturbance conditions, and were resilient within ten to twenty years after the stressor subsides. We have no reason to suspect that temperate soils in Centralia are exceptional as compared to other soils. Thus, these results suggest that soils may have an intrinsic capacity for robustness to varied disturbances, even to those disturbances considered to be ‘extreme', compounded, or incongruent with natural conditions. Understanding the precise functional underpinnings of soil microbial community resilience, including the roles of seed banks in determining that resilience, is a next important step in predicting and, potentially, managing, microbial community responses to disturbances.

## Figures and Tables

**Table 1:** Ten most abundant OTUs in fire-affected Centralia soils

*OTU ID*	*Cumulative % abundance (out of total no. sequences in fire-affected samples)*	*% occurrence (out of 9 warm or venting fire-affected soils)*	*Taxonomic assignment*
111933	5.5%	100%	Archaea; Crenarchaeota; MBGA
OTU_dn_1	2.5	100%	Bacteria; Chloroflexi; Ktedonobacteria;Thermogemmatisporales; Thermogemmatisporaceae;
OTU_dn_2	2.2	100%	Bacteria; Chloroflexi; Ktedonobacteria;Thermogemmatisporales Thermogemmatisporaceae
242467	2.0	100%	Bacteria; Acidobacteria; DA052;Ellin6513
174835	2.0	100%	Archaea; Crenarchaeota; Thermoprotei;YNPFFA; SK322
61819	1.7	100%	Bacteria; Acidobacteria; TM1
OTU_dn_17	1.5	78%	Bacteria; Proteobacteria; Deltaproteobacteria
215700	1.4	100%	Bacteria; Acidobacteria; Acidobacteriia;Acidobacteriales; Koribacteraceae
OTU_dn_8	1.3	100%	Bacteria
OTU_dn_3	1.2	100%	Bacteria

Abbreviation: OTU, Operational Taxonomic Unit.

OTUs (defined at 97% sequence identity) were assigned to the most resolved taxonomic level possible; there were no taxonomic assignments that could be made to these prevalent OTUs below the family level (RDP Classifier confidence>0.80).
