# Supplementary material for: Divergent extremes but convergent recovery of bacterial and archaeal soil communities to an ongoing subterranean coal mine fire
Source: ISME J. 2017 Mar 10;11(6):1447–59. doi: 10.1038/ismej.2017.1 (PMC5437352; doi:10.1038/ismej.2017.1)
Supplement: Supplementary Materials [file ismej20171x1.docx]

*Supplementary Materials for*

Divergent extremes but convergent recovery of bacterial and archaeal soil communities to an ongoing subterranean coal mine fire

Sang-Hoon Lee, Jackson W Sorensen, Keara L Grady, Tammy C Tobin, and Ashley Shade

**Supplementary Methods: Quantitative PCR**

We performed quantitative PCR (qPCR) using bacterial and archaeal 16S rRNA gene universal primer sets (**Supplementary Table 1;** Caporaso *et al.*, 2012). The qPCR was conducted in 20 µL reactions, consisting of 10 µL SYBR qPCR Master mix (Quanta Bioscience, Gaithersburg, MD, USA), 0.4 pM each of the forward and the reverse primers, and 2 µL of template DNA. Triplicate qPCR reactions for each DNA sample was performed. The thermal profile was as follows: initial denaturation at 95^o^C for 10 s, followed by 40 cycles of denaturation at 95^o^C for 10 s, annealing at 50^o^C for 15 s, and extension at 72^o^C for 40 s. A final dissociation protocol (58^o^C to 94.5^o^C, increment 0.5^o^C for 10 s) was performed to ensure the absence of nonspecific amplicons. The reactions were conducted using the Bio-Rad iQ5 real time detection system (Bio-Rad, Hercules, CA, USA).

To create the standard curve for the primer set, extracted *E. coli* K-12 MG1655 genomic DNA was used to amplify 16S rRNA genes with the 515F and 806R universal primer set (Caporaso *et al.* 2012). The reaction mixtures consisted of 1X final concentration GoTaq® Green Master Mix (Promega), 1 pM each of the forward and the reverse primers, and 1 µL of *E. coli* template DNA, in a 50 µL final volume. The thermal profile was as follows: initial denaturation at 95^o^C for 10 s, followed by 30 cycles of denaturation at 95^o^C for 10 s, annealing at 50^o^C for 15 s, and extension at 72^o^C for 40 s. Amplified *E.coli* PCR products were purified using Promega Wizard SV Gel and PCR Cleanup System per manufacturer’s instructions. Purified PCR amplicons were cloned into the TOPO cloning vectors with a TOPO TA cloning kit (Invitrogen, Carlsbad, Calif.) according to the manufacturer’s protocol. Cloned plasmid DNA was extracted using QIAPrep Spin Plasmid Miniprep kit (Qiagen) following manufacturer’s protocol, and the concentration was measured using Qubit® dsDNA BR Assay Kit (Life Technologies, NY, USA). A standard curve was then constructed using a 10-fold dilution series of cloned plasmid DNA. Based on the DNA size for plasmid DNA clone and Avogadro’s number (6.02 x 10^23^ molecules per mole), we calculated the copy number of cloned plasmid DNA (where 4.52 x 10^-3^ fg is equal to one plasmid copy). qPCR amplifications were performed in triplicate with a range of concentrations from 18.8 to 1.88 x 10^8^ copies of plasmid DNA using Bio-Rad iQ5 real time detection system, and the observed C_T_ values were plotted with regression curve using Sigma plot software (**Supplementary Figure 8**). Copy number of 16S rRNA genes in each DNA sample was determined based on the observed C_T_ values calculated by function of regression curve [Y = -3.13x + 41.81, where x is observed C_T_ value and Y is converted copy number of 16S rRNA gene. The qPCR efficiency, *E*, was calculated based on the slope in the qPCR standard curves as described by Rasmussen 2001:

$$E={10}^{\left[ \frac{-1}{slope} \right]}$$

According to this calculation, the qPCR amplification efficiency of 16S rRNA gene using EMP primers was 2.08.

To calculate 16S rRNA copies per gram of dry soil, the average copies of the three qPCR technical replicates per DNA extraction was multiplied by the dilution factor (the elution volume of the DNA extraction divided by the microliters added to the qPCR reaction), and then that value was divided by the dry mass of the soil used for the DNA extraction to get copies per gram of dry soil.

**Supplementary Results: Sequencing summary**

After quality filtering, our 16S rRNA amplicon dataset produced 5,778,000 high-quality reads (5,776,626 sequences after omitting singletons OTUs) with a UPARSE-calculated error rate of 0.469%. In total, we observed 28,220 OTUs (26,846 when omitting singleton OTUs) defined at 97% sequence identity; approximately one-third of OTUs were defined based on high-identity matches to the greengenes v13.8 reference database (8,967 OTUs; 8,794 when omitting singleton OTUs), while two-thirds were defined *de novo* after unsuccessful attempts to match the database (19,253 OTUs; 18,052 when omitting singleton OTUs). We observed 65 phyla in Centralia soils.

Though it was not unexpected in a soil ecosystem impacted by an unusual disturbance, the observation of a large proportion on *de novo* OTUs (with the open-reference OTU picking workflow) suggests that Centralia soils may harbor substantial undescribed microbial diversity and functions. Coal mine fire ecosystems have been sources of novel microbial functions, including reported aerobic nitrogen fixation (Ribbe et al. 1997) and novel antibiotics (Wang et al. 2014a, 2014b). Furthermore, thermophiles are of interest for bioprospecting for natural products such as thermally-stable enzymes (e.g., for biomass deconstruction from lignocellulosic crops (Blumer-Schuette et al. 2014) and novel antibiotics (Garg et al. 2012). Among the *de novo* lineages of interest were several archaeal taxa tentatively identified as Crenarcheaota and Parvarcheaota, and several minor bacterial lineages tentatively assigned as TM6, TM7, OD1, OP11, LD1, WPS-2, and WS-3. A 16S rRNA clone library and T-RFLP study of three soil microbial communities that were each proximate to active coal seam vents in China also reported a proportionally large number of Crenarcheaota among detected archaeal clones (Zhang et al. 2013), suggesting that these may be common inhabitants of soils impacted by long-term fires.

*Supplementary references*

Blumer-Schuette, S. E., S. D. Brown, K. B. Sander, E. A. Bayer, I. Kataeva, J. V. Zurawski, J. M. Conway, M. W. W. Adams, and R. M. Kelly. 2014. Thermophilic lignocellulose deconstruction. FEMS Microbiology Reviews 38:393–448.

Caporaso, J. G., C. L. Lauber, W. a Walters, D. Berg-Lyons, J. Huntley, N. Fierer, S. M. Owens, J. Betley, L. Fraser, M. Bauer, N. Gormley, J. a Gilbert, G. Smith, and R. Knight. 2012. Ultra-high-throughput microbial community analysis on the Illumina HiSeq and MiSeq platforms. The ISME Journal 6:1621–1624.

Garg, N., W. Tang, Y. Goto, S. K. Nair, and W. a. van der Donk. 2012. Lantibiotics from Geobacillus thermodenitrificans. Proceedings of the National Academy of Sciences of the United States of America 109:5241–5246.

Rasmussen, R. 2001. Quantification on the LightCycler. Rapid Cycle Real-Time PCR: Methods and Applications:p21–34. Springer Berlin Heidelberg

Ribbe, M., D. Gadkari, and O. Meyer. 1997. N2 Fixation by Streptomyces thermoautotrophicus Involves a Molybdenum-Dinitrogenase and a Manganese-Superoxide Oxidoreductase That Couple N2Reduction to the Oxidation of Superoxide Produced from O2by a Molybdenum-CO Dehydrogenase. Journal of Biological Chemistry 272:26627–26633.

Wang, X., S. I. Elshahawi, K. A. Shaaban, L. Fang, L. V. Ponomareva, Y. Zhang, G. C. Copley, J. C. Hower, C. G. Zhan, M. K. Kharel, and J. S. Thorson. 2014a. Ruthmycin, a new Tetracyclic Polyketide from Streptomyces sp. RM-4-15. Organic Letters 16:456–459.

Wang, X., K. A. Shaaban, S. I. Elshahawi, L. V Ponomareva, M. Sunkara, G. C. Copley, J. C. Hower, A. J. Morris, M. K. Kharel, and J. S. Thorson. 2014b. Mullinamides A and B, new cyclopeptides produced by the Ruth Mullins coal mine fire isolate Streptomyces sp. RM-27-46. The Journal of antibiotics 67:571–5.

Zhang, T., J. Xu, J. Zeng, and K. Lou. 2013. Diversity of prokaryotes associated with soils around coal-fire gas vents in MaNasi county of Xinjiang, China. Antonie van Leeuwenhoek, International Journal of General and Molecular Microbiology 103:23–36.

**Supplementary Figures**

**Supplementary Figure 1.** Soil sampling sites at Centralia mine fire. In total, 18 surface soil samples (5.08 cm x 20 cm PVC core) were collected along two fire fronts in Centralia, on 15/16 October 2014. Sampling sites encompass a gradient of historical fire activity (red flags: Fire-affected in 2014 (temperature > 21°C); yellow flags: recovered in temperature, post-fire; and green flags: reference soils).

**Supplementary Figure 2**. PCoA showing the variability among technical replicates. Three replicate DNA extractions, amplifications and sequencing reactions were performed per soil, and these sequences were subsequently pooled into one aggregate set of sequences to achieve deep coverage of the community within each soil. Error bars are standard deviation around the mean weighted UniFrac distance among technical replicates, each subsampled to an even 53,000 sequences per replicate.

**Supplementary Figure 3.** Soil physical and chemical contextual data (x-axis) plotted against temperature (y-axis). Color gradient shows the soil temperature, and symbols show soil fire classification in October 2014 as fire-affected, recovered, or reference.

**Supplementary Figure 4**. Quantification of (**A**) 16S rRNA copies per gram of dry soil and (**B**) cell counts per gram of dry soil in fire-affected, recovered, and reference soils. 16S rRNA copies were assessed using quantitative PCR, and cell counts were assessed using cell separation from soil, staining and microscope imaging. There were no statistical differences in values across fire classification for either measurement (all pairwise p > 0.09 with a student’s t-test).

**Supplementary Figure 5**. Centralia 16S rRNA amplicon sequencing effort assessed by subsampling/rarefaction of (**A**) richness and (**B**) Faith’s phylogenetic diversity with increasing total number of sequences.

**Supplementary Figure 6**. Divergences in fire-affected soils are not well explained by temperature. **(A)** Principal coordinate analysis (PCoA) based on weighted UniFrac distances of phylogenetic bacterial and archaeal community structure in fire-affected soils. The strength of statistically significant (p < 0.10) explanatory variables are shown with blue arrows. **(B)** Constrained analysis (CAP) based on weighted UniFrac distances, where the explanatory value of temperature is removed from the analysis to understand the influence of the remaining explanatory variables.

**Supplementary Figure** **7.** Neutral models of community assembly (abundance v. occurrence) for (**A**) the total community (“All”, n= 18), **(B)** recovered soils (“Recovered” n=7), and **(C)** fire-affected soils (“Fire_Affected”, n=9). Red symbols show OTUs that had higher abundance than their prediction, and blue symbols show OTUs that had lower abundance than their prediction. The thick yellow line is the neutral model prediction, and the thin yellow lines show a 95% confidence interval around the prediction.

**
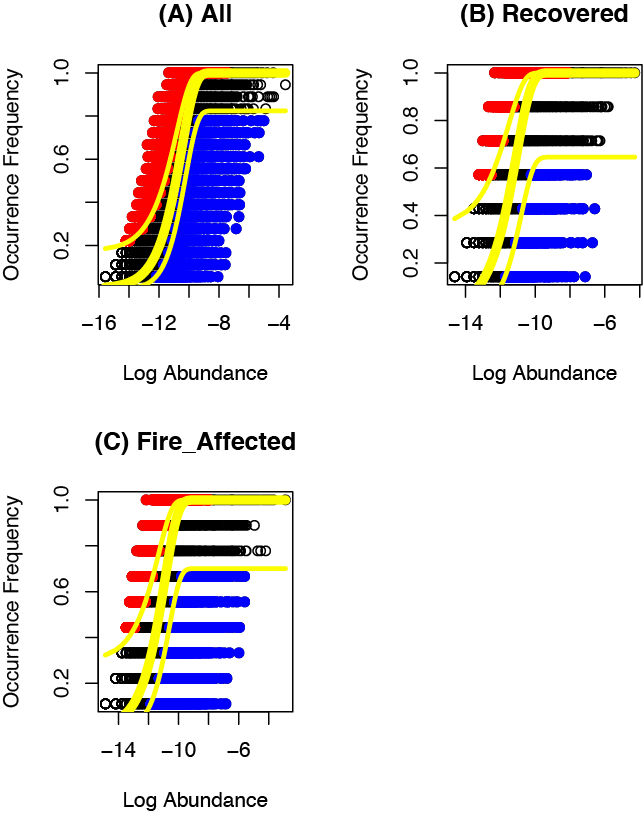
**

**Supplementary Figure 8**. **­­­** Quantitative PCR standard curve for the amount of *E.coli* 16S rRNA gene copies (cloned into plasmids) versus C_T_ values. The solid line is the regression (R^2^ = 0.988). The error bars are the standard deviations obtained in three independent experiments.


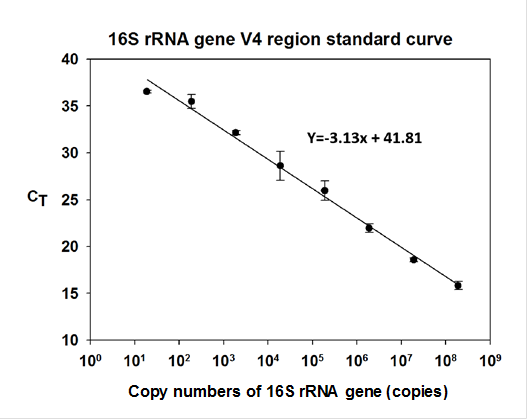


**Supplementary Table 1.** Primers used in this study.

|  |  |  |  |  |  |  |
| --- | --- | --- | --- | --- | --- | --- |
| **Table 1.** Primer set used for this study. | |  |  |  |  |  |
| Primer name | sequence (5' - 3') | Target | target site | Product size | Tm | Reference |
|  |  |  |  | (bp) |  |  |
| 515F | GTGCCAGCMGCCGCGGTAA | 16S V4 | 515-534 | 291 | 69.5 | Caporaso et al., ISME J. 2012 |
| 806R | GGACTACHVGGGTWTCTAAT |  | 787-806 |  | 45.1 |  |

**Supplementary Table 2**. Mean and standard deviation (“sd”) of phylogenetic diversity and number of OTUs (“richness) across technical sequencing replicates for the un-collapsed dataset (rarefied to 53,000 sequences per sample). Three replicate DNA extractions, amplifications and sequencing reactions were performed per soil, and, after calculating the technical variability, these sequences were pooled into one aggregate set of sequences to achieve deep coverage of the community within each soil.

| SampleID | PD_mean | PD_sd | Richness_mean | Richness_sd |
| --- | --- | --- | --- | --- |
| C01 | 393.96 | 16.22 | 4073.67 | 55.77 |
| C02 | 392.48 | 9.42 | 3805.00 | 48.50 |
| C03 | 403.12 | 15.25 | 4498.67 | 39.72 |
| C04 | 374.95 | 6.51 | 4420.33 | 89.51 |
| C05 | 405.05 | 14.17 | 4389.33 | 109.25 |
| C06 | 332.89 | 13.26 | 3718.67 | 117.33 |
| C07 | 371.50 | 7.80 | 4253.00 | 67.01 |
| C08 | 525.93 | 5.37 | 6011.67 | 191.04 |
| C09 | 312.71 | 32.40 | 2328.33 | 352.23 |
| C10 | 267.32 | 27.06 | 2128.00 | 225.08 |
| C11 | 343.84 | 12.26 | 3886.67 | 81.56 |
| C12 | 249.92 | 29.65 | 2106.67 | 280.73 |
| C13 | 316.18 | 58.27 | 2471.00 | 816.28 |
| C14 | 307.29 | 16.47 | 2688.67 | 232.20 |
| C15 | 330.40 | 38.06 | 3011.67 | 435.15 |
| C16 | 356.85 | 12.24 | 3546.33 | 83.93 |
| C17 | 506.13 | 19.77 | 5724.00 | 179.43 |
| C18 | 392.64 | 13.98 | 4210.67 | 105.61 |

**Supplementary Table 3.** (A) Percent variation explained for PCoA axes 1 and 2 for weighted and unweighted UniFrac, Sorensen-dice, and Bray-Curtis distances/dissimilarities. Nonnormalized Weighted UniFrac was chosen because it was most informative in explaining the variance along the first two axes. (B) Pairwise resemblance correlations calculated with Mantel and PROTEST. All p < 0.001 for all tests.

A.

|  | PCoA1 | PCoA2 |
| --- | --- | --- |
| Weighted UniFrac | 77.1 | 12.7 |
| Normalized Weighted Unifrac | 74.6 | 10.9 |
| Unweighted UniFrac | 18.3 | 13.6 |
| Sorensen-dice | 20.1 | 15.2 |
| Bray-Curtis | 23.9 | 13.7 |

B.

| **Dist1** | **Dist2** | **Mantel_R** | **PROTEST_R** | **PROTEST_m12** |
| --- | --- | --- | --- | --- |
| weighted_UniFrac | unweighted_UniFrac | 0.63 | 0.67 | 0.55 |
| weighted_UniFrac | normalized_weighted_UniFrac | 0.96 | 0.98 | 0.03 |
| weighted_UniFrac | BrayCurtis | 0.72 | 0.76 | 0.42 |
| weighted_UniFrac | Sorenson | 0.68 | 0.71 | 0.50 |
| unweighted_UniFrac | normalized_weighted_UniFrac | 0.61 | 0.69 | 0.52 |
| unweighted_UniFrac | BrayCurtis | 0.81 | 0.95 | 0.09 |
| unweighted_UniFrac | Sorensen | 0.94 | 0.99 | 0.02 |
| normalized_weighted_UniFrac | BrayCurtis | 0.70 | 0.78 | 0.39 |
| normalized_weighted_UniFrac | Sorensen | 0.69 | 0.73 | 0.47 |
| BrayCurtis | Sorensen | 0.85 | 0.97 | 0.06 |

**Supplementary Table 4**. Explanatory value of soil contextual data to changes in Centralia soil community structure along PCoA axes for the all soils. Factors significant at p < 0.10 are in bold.

|  | PCoA1 | PCoA2 | R2 | P value |  |
| --- | --- | --- | --- | --- | --- |
| % explanation | 77.1 | 12.7 |  |  |  |
| **Soil Temperature** | **0.968** | **-0.252** | **0.787** | **0.002** | ****** |
| **NO_3_N (ppm)** | **0.226** | **-0.974** | **0.290** | **0.067** | **.** |
| **pH** | **0.185** | **0.983** | **0.649** | **0.008** | ****** |
| K (ppm) | -0.813 | 0.582 | 0.006 | 0.946 |  |
| Mg (ppm) | -0.148 | 0.989 | 0.123 | 0.374 |  |
| Organic matter | 0.812 | -0.583 | 0.002 | 0.984 |  |
| **NH_4_N (ppm)** | **0.194** | **-0.981** | **0.287** | **0.088** | . |
| SulfateSulfur (ppm) | 0.121 | -0.993 | 0.116 | 0.372 |  |
| **Ca (ppm)** | **0.182** | **0.983** | **0.529** | **0.022** | ***** |
| **Fe (ppm)** | **0.253** | **-0.967** | **0.271** | **0.094** | **.** |
| Fire history | -0.605 | 0.797 | 0.253 | 0.169 |  |
| As (ppm) | -0.014 | -1.000 | 0.124 | 0.404 |  |
| P (ppm) | 0.435 | -0.900 | 0.093 | 0.462 |  |
| **Soil Moisture (%)** | **0.263** | **-0.965** | **0.405** | **0.035** | ***** |
| Significant codes: ‘***’ 0.001; ‘**’ 0.01; ‘*’ 0.05; ‘.’ 0.1; ‘ ’ 1 | | | | | |
| Number of permutations: 999 | | | | | |

**Supplementary Table 5**. Explanatory value of soil contextual data to changes in Centralia soil community structure along PCoA axes for the fire-affected soils. Factors significant at p < 0.10 are in bold.

|  | PCoA1 | PCoA2 | R2 | P value |  |
| --- | --- | --- | --- | --- | --- |
| % explanation | 70.9 | 22.0 |  |  |  |
| **SoilTemperature_to10cm** | **0.765** | **-0.644** | **0.578** | **0.088** | **.** |
| NO3N_ppm | -0.002 | -1.000 | 0.328 | 0.236 |  |
| **pH** | **0.490** | **0.872** | **0.823** | **0.002** | ****** |
| K_ppm | 0.282 | -0.959 | 0.232 | 0.429 |  |
| **Mg_ppm** | **0.767** | **0.641** | **0.604** | **0.058** | **.** |
| OrganicMatter_500 | 0.407 | -0.913 | 0.218 | 0.498 |  |
| NH4N_ppm | -0.021 | -1.000 | 0.342 | 0.155 |  |
| SulfateSulfur_ppm | -0.216 | -0.976 | 0.118 | 0.759 |  |
| **Ca_ppm** | **0.613** | **0.790** | **0.694** | **0.015** | ***** |
| Fe_ppm | 0.044 | -0.999 | 0.355 | 0.204 |  |
| As_ppm | -0.492 | -0.871 | 0.388 | 0.228 |  |
| P_ppm | 0.142 | -0.990 | 0.238 | 0.453 |  |
| SoilMoisture_Per | -0.023 | -1.000 | 0.460 | 0.143 |  |
| Fire_history | 0.742 | -0.670 | 0.136 | 0.637 |  |
| Significant codes: ‘***’ 0.001; ‘**’ 0.01; ‘*’ 0.05; ‘.’ 0.1; ‘ ’ 1 | | | | | |
| Number of permutations: 999 | | | | | |

**Supplementary Table 6**. Explanatory value of soil contextual data to changes in Centralia soil community structure along the constrained PCoA axes for the fire-affected soils, after removing the influence of temperature. Factors significant at p < 0.10 are in bold.

|  | CAP_A1 | CAP_A2 | R2 | P value |  |
| --- | --- | --- | --- | --- | --- |
| % explanation | 64.2 | 25.9 |  |  |  |
| SoilTemperature_to10cm | 1.000 | 0.000 | 0.000 | 1.000 |  |
| NO3N_ppm | -0.973 | -0.233 | 0.354 | 0.285 |  |
| **pH** | **0.771** | **0.637** | **0.729** | **0.014** | ***** |
| K_ppm | -0.416 | -0.909 | 0.093 | 0.730 |  |
| Mg_ppm | 0.641 | 0.767 | 0.370 | 0.247 |  |
| OrganicMatter_500 | 0.070 | -0.997 | 0.128 | 0.613 |  |
| NH4N_ppm | -0.962 | -0.273 | 0.367 | 0.240 |  |
| SulfateSulfur_ppm | -0.988 | 0.154 | 0.234 | 0.446 |  |
| **Ca_ppm** | **0.652** | **0.759** | **0.551** | **0.092** | **.** |
| Fe_ppm | -0.862 | -0.508 | 0.396 | 0.355 |  |
| As_ppm | -0.948 | -0.317 | 0.378 | 0.216 |  |
| P_ppm | -0.132 | -0.991 | 0.287 | 0.350 |  |
| SoilMoisture_Per | -0.813 | -0.583 | 0.419 | 0.203 |  |
| Fire_history | 0.636 | -0.771 | 0.276 | 0.375 |  |
| Significant codes: ‘***’ 0.001; ‘**’ 0.01; ‘*’ 0.05; ‘.’ 0.1; ‘ ’ 1 | | | | | |
| Number of permutations: 999 | | | | | |
|  | | | | | |

**Supplementary Table 7**. Parameters and fits of neutral models as per Burns et al. 2015.

| *Model parameter* | *all* | *Fire-affected* | *Recovered* |
| --- | --- | --- | --- |
| **m** | 0.04 | 0.08 | 0.10 |
| **m.ci** | 0.00 | 0.00 | 0.00 |
| **m.mle** | 0.04 | 0.08 | 0.10 |
| **maxLL** | -5838.12 | 1187.68 | -2735.42 |
| **binoLL** | 475.69 | 1162.47 | -143.93 |
| **poisLL** | 475.67 | 1162.46 | -143.94 |
| **Rsqr** | 0.45 | 0.12 | 0.53 |
| **Rsqr.bino** | -1.19 | -0.86 | -0.47 |
| **Rsqr.pois** | -1.19 | -0.86 | -0.47 |
| **RMSE** | 0.20 | 0.26 | 0.21 |
| **RMSE.bino** | 0.39 | 0.38 | 0.37 |
| **RMSE.pois** | 0.39 | 0.38 | 0.37 |
| **AIC** | -11672.24 | 2379.36 | -5466.85 |
| **BIC** | -11655.75 | 2394.86 | -5451.16 |
| **AIC.bino** | 955.38 | 2328.94 | -283.86 |
| **BIC.bino** | 971.88 | 2344.43 | -268.17 |
| **AIC.pois** | 955.35 | 2328.92 | -283.88 |
| **BIC.pois** | 971.84 | 2344.42 | -268.19 |
| **N** | 321000.00 | 321000.00 | 321000.00 |
| **Samples** | 18.00 | 9.00 | 7.00 |
| **Richness** | 28220.00 | 17097.00 | 18866.00 |
| **Detect** | 0.00 | 0.00 | 0.00 |
| **%AbovePred** | 0.14 | 0.12 | 0.13 |
| **%BelowPred** | 0.10 | 0.07 | 0.12 |

**Supplementary Table 8**. Welch’s t-tests comparing the mean relative abundances of phyla across fire-affected and recovered soils. Bold values are significant at p < 0.05.

| Phylum | T-statistic | DF | p-value |
| --- | --- | --- | --- |
| Crenarchaeota | **2.80** | **8.36** | **0.02** |
| Euryarchaeota | -0.47 | 11.86 | 0.65 |
| [Parvarchaeota] | **-3.31** | **11.34** | **0.01** |
| Unidentified Bacteria | **2.33** | **8.22** | **0.05** |
| AD3 | -1.58 | 7.28 | 0.16 |
| Acidobacteria | -1.74 | 13.64 | 0.10 |
| Actinobacteria | -0.22 | 13.12 | 0.83 |
| Armatimonadetes | -0.58 | 13.21 | 0.57 |
| Bacteroidetes | **-4.00** | **9.73** | **0.00** |
| Chlamydiae | -1.68 | 10.73 | 0.12 |
| Chlorobi | -0.43 | 10.96 | 0.67 |
| Chloroflexi | **2.82** | **9.67** | **0.02** |
| Cyanobacteria | 1.85 | 8.07 | 0.10 |
| Elusimicrobia | **-3.45** | **8.01** | **0.01** |
| FCPU426 | -0.79 | 11.28 | 0.45 |
| Firmicutes | 0.60 | 10.97 | 0.56 |
| Gemmatimonadetes | **-2.24** | **12.33** | **0.04** |
| Nitrospirae | 0.04 | 12.47 | 0.97 |
| OD1 | -1.28 | 10.05 | 0.23 |
| OP11 | -1.82 | 7.56 | 0.11 |
| Planctomycetes | **-3.33** | **11.61** | **0.01** |
| Proteobacteria | **-2.42** | **12.89** | **0.03** |
| SBR1093 | 2.02 | 8.00 | 0.08 |
| Spirochaetes | **-2.43** | **6.68** | **0.05** |
| TM6 | **-2.48** | **7.47** | **0.04** |
| Tenericutes | 0.14 | 10.06 | 0.89 |
| Verrucomicrobia | **-3.78** | **10.92** | **0.00** |
| WPS-2 | 0.41 | 10.37 | 0.69 |
| WS3 | -2.26 | 6.59 | 0.06 |
| Below_0.01 | -0.27 | 8.39 | 0.79 |
